# Arsenic in the Human Body

**Published:** 1902-06-14

**Authors:** 


### ARSENIC IN THE HUMAN BODY.

One result of recent outbreaks of arsenical poisoning arising from the presence of undue quantities of arsenic in articles of diet, and more particularly in beer, has been to lead to a search for very minute quantities of this metal, and one result of this search has been to discover it in small but appreciable quantities in many unexpected places. It would seem, indeed, that a certain quantity of arsenic is to be regarded as normal to the tissues of the human body. According to recent investigations, however, it appears that the metal is not generally diffused throughout the body but is practically concentrated in the thyroid gland. A very small quantity also occurs in the thymus, while traces are found in the skin, hair, and nails, and also in the bones and brain. But other parts of the body are said to be free from it. We will not here speculate as to the meaning of all this, but it is a matter of considerable interest that arsenic—such a profound modifier of nutrition—should be found to be concentrated so largely upon one organ which itself also has so great an influence upon nutrition as the thyroid has been shown to have.

